# 646. Clinical and microbiological outcomes of omadacycline for pulmonary *Mycobacterium abscessus complex*

**DOI:** 10.1093/ofid/ofae631.211

**Published:** 2025-01-29

**Authors:** Mohammed Al Musawa, Raaga Vemula, Mehriban Mammadova, Carly Wadle, Anahit Muscarella, Christo Cimino, John Zeuli, Catessa A Howard, Saira Butt, Carlos Mejia-Chew, Yasir Hamad, Aaron Ong, Keira A Cohen, Emily A Kaip, Maria G Tupayachi-Ortiz, Christina T Fiske, Chloe Judd, Kaylee E Caniff, Michael J Rybak

**Affiliations:** Wayne State University, Detroit, Michigan; University of Texas at Tyler, Tyler, Texas; University of Texas Health Science Center, Houston, Texas; University of Texas Health Science Center, Houston, Texas; Vanderbilt University Medical Center, Nashville, Tennessee; Vanderbilt University Medical Center, Nashville, Tennessee; Mayo Clinic, Rochester, Minnesota; West Virginia University Medicine, Morgantown, West Virginia; Indiana University School of Medicine, Indianapolis, Indiana; Washington University in St Louis, St. Louis, Missouri; Washington University, Herndon, VA; Johns Hopkins University School of Medicine, Baltimore, Maryland; Johns Hopkins University School of Medicine, Baltimore, Maryland; University of California, San Francisco Medical Center, San Francisco, California; Department of Medicine, Division of Pulmonary and Critical Care Medicine, Miller School of Medicine, University of Miami, Miami, Florida; Vanderbilt University Medical Center, Nashville, Tennessee; Wayne State University, Detroit, Michigan; Anti-Infective Research Lab, Eugene Applebaum College of Pharmacy and Health Sciences, Wayne State University, Royal Oak, Michigan; Eugene Applebaum College of Pharmacy and Health Sciences, Detroit, Michigan

## Abstract

**Background:**

*Mycobacterium abscessus* complex (MABC) is a difficult-to-treat infection due to antibiotic resistance. Our study aimed to assess omadacycline’s (OMC) clinical and microbiological outcomes for the treatment of pulmonary MABC.

**Figure:**
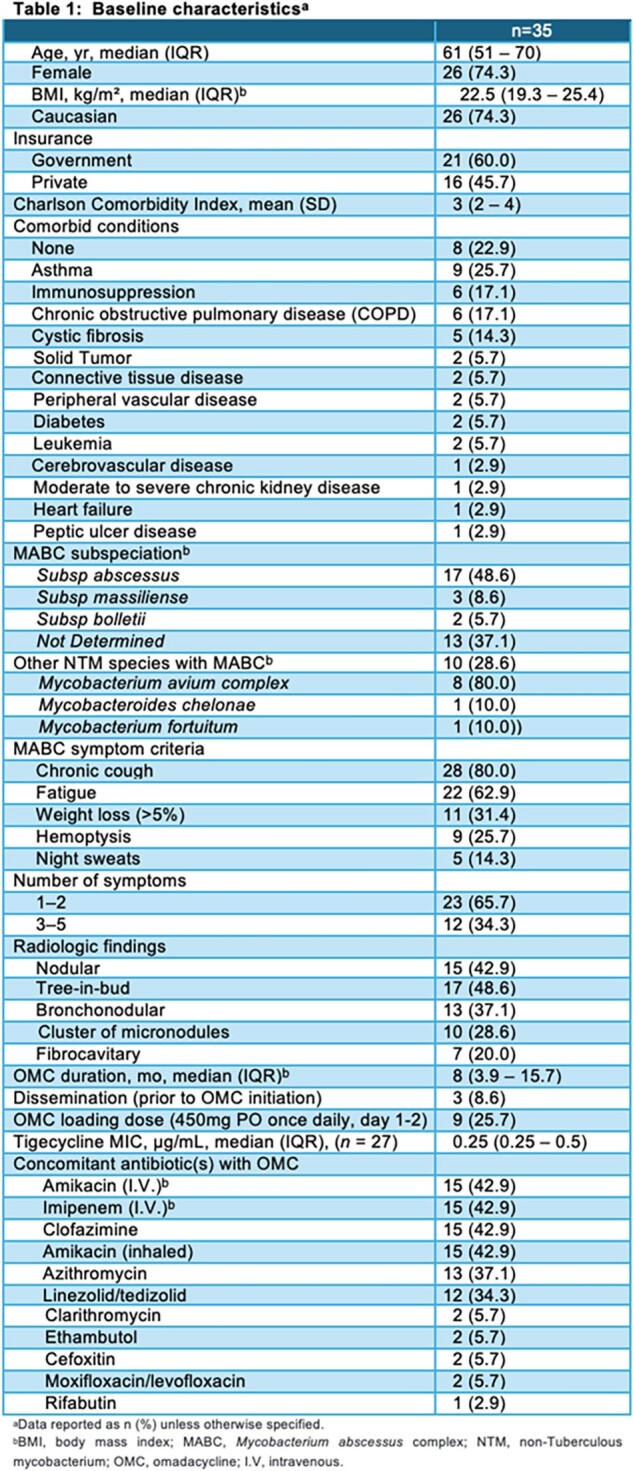

**Methods:**

A retrospective study was carried out across 12 US medical institutions from 1/2018-4/2023 to examine the clinical outcomes, and tolerability of OMC treatment for pulmonary MABC. Patients aged ≥ 18 years who were treated with OMC for ≥ 3 months were included. The primary outcome was clinical success at 3, 6, and 12 months. The secondary outcomes were sputum culture conversion rate, adverse effects, and clinical success by subspecies and macrolide susceptibility.

**Figure:**
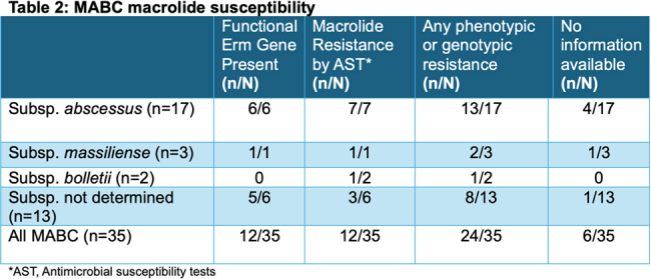

**Results:**

Thirty-five patients were included in this analysis. Most patients were female (74.3%) and Caucasian (74.3%), with a median (IQR) age of 61 years (51–70). Subspeciation was performed for 22 isolates with predominant *M. abscessus* subspecies (77.3%). Moreover, coinfection with other NTM species was present in 28.6% of cases where *Mycobacterium avium* complex was present in 8 cultures. Sixty-eight percent of the MABC isolates were confirmed to be macrolide resistance; half (12/24, 50.0%) were evident by the presence of functional *erm* gene, while the other half by antimicrobial susceptibility (Table 3). Of the remaining isolates, 14% were macrolide-susceptible, while no information was reported in 17%. The median (IQR) treatment duration of OMC was 8 months (3.9 – 15.7). The most commonly co-administered antibiotics were intravenous amikacin, imipenem/cilastatin, inhaled amikacin and clofazimine with the same percentage (42.9%) (Table 1). Overall, MABC clinical success was observed in 71.4%, 89.7%, and 90.9% in 3-, 6- and 12 months, respectively (Table 3). Adverse effects reported in 34.3%. The most common side effects were gastrointestinal intolerance (25.7%) and hepatoxicity (11.4%), which led to drug discontinuation in 22.9%.

**Figure:**
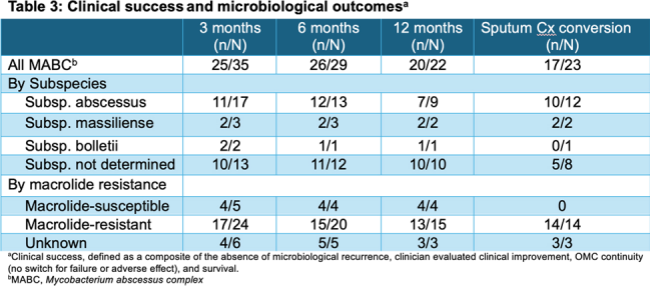

**Conclusion:**

OMC treatment showed clinical success in > 70% of patients with pulmonary MABC including patients with macrolide resistant strains for more than 3 months. However, larger studies are needed to validate the outcomes beyond 12 months.

**Disclosures:**

**Kaylee E. Caniff, PharmD, BCIDP**, T2Biosystems: Honoraria **Michael J. Rybak, PharmD, PhD, MPH**, Abbvie, Melinta, Sionogi, Merck, T2Biosystems: Advisor/Consultant|Abbvie, Melinta, Sionogi, Merck, T2Biosystems: Grant/Research Support|Abbvie, Melinta, Sionogi, Merck, T2Biosystems: Speaker

